# Sirtuins and their Biological Relevance in Aging and Age-Related Diseases

**DOI:** 10.14336/AD.2019.0820

**Published:** 2020-07-23

**Authors:** Lijun Zhao, Jianzhong Cao, Kexin Hu, Xiaodong He, Dou Yun, Tanjun Tong, Limin Han

**Affiliations:** ^1^Peking University Research Center on Aging, Department of Biochemistry and Molecular Biology, School of Basic Medical Sciences, Peking University Health Science Center, Beijing Key Laboratory of Protein Posttranslational Modifications and Cell Function, Beijing, China; ^2^Department of General Surgery, Peking Union Medical College Hospital, Chinese Academy of Medical Sciences & Peking Union Medical College, Beijing, China.

**Keywords:** sirtuins, aging, age-related diseases, cancer

## Abstract

Sirtuins, initially described as histone deacetylases and gene silencers in yeast, are now known to have many more functions and to be much more abundant in living organisms. The increasing evidence of sirtuins in the field of ageing and age-related diseases indicates that they may provide novel targets for treating diseases associated with aging and perhaps extend human lifespan. Here, we summarize some of the recent discoveries in sirtuin biology that clearly implicate the functions of sirtuins in the regulation of aging and age-related diseases. Furthermore, human sirtuins are considered promising therapeutic targets for anti-aging and ageing-related diseases and have attracted interest in scientific communities to develop small molecule activators or drugs to ameliorate a wide range of ageing disorders. In this review, we also summarize the discovery and development status of sirtuin-targeted drug and further discuss the potential medical strategies of sirtuins in delaying aging and treating age-related diseases.

In 1979, the discovery of mating-type regulator 1 (MAR1) in *Saccharomyces cerevisiae* was reported [1]. Lack of this protein resulted in the inhibition of silencing of HM loci, which control mating type and sterility in yeast. Three more proteins with similar functions were discovered later in 1979, and the nomenclature was unified, thus creating a family of silent information regulator proteins, *Sirs* [2]. Emerging interest in sirtuins occurred in 1999 when it was reported that *Sir2* overexpression could extend yeast lifespans by as much as 70% [3]. Further research revealed that sirtuins overexpression also leads to lifespan extension in other model organisms, such as *Caenorhabditis elegans* and *Drosophila melanogaster.*

Shortly thereafter, it was shown that sirtuins appear to be conserved from yeast to mammals, however the complexity of their function increases with the complexity of the organism [4]. In yeast, in addition to the chief representative, *Sir2*, there are four more homologous proteins [2], and the positive effect of sirtuins activity can be attributed to the increase in genomic stability. In mammals there are seven enzymes belonging to the sirtuin family, *sirt1-7*. Phylogenetic analysis of 60 core domains from different eukaryotes and prokaryotes places the mammalian sirtuins into four different classes (I—IV). *Sirt1, sirt2* and *sirt3* are known as Class I sirtuins, which groups all yeast sirtuins and also at least one of the *sir2*-related proteins in most eukaryotes. Class I is divided in three sub-classes: a, b and c. *Sirt1* belongs to Class Ia which also includes *Sir2* and *Hst1* from S. cerevisiae, *C. elegans Sir*-2.1 and *D. melanogaster* D. mel1. *Sirt2* and *sirt3* reside in Class Ib, together with yeast *Hst2*, fly D. mel2 and other fungi and protozoa sirtuins. *Sirt4* is part of Class II, which also includes sirtuins from bacteria, insects, nematodes, mould fungus and protozoans. *Sirt5* is the mammalian member of Class III sirtuins, distributed widely in all prokaryotes either bacteria or archaea. Finally, Class IV contains *sirt6* and *sirt7* in two different sub-classes IVa and IVb respectively; and unlike Class III, sirtuins of this class are not present in prokaryotes, but are broadly distributed in metazoans, plants and vertebrates [5].

In addition, mammalian sirtuins also differ in their sub-cellular localization, and some sirtuins can relocalize depending on the cell or tissue type, the developmental stage, metabolic status, and certain stress conditions. *Sirt1* is localized to the nucleus [6], but it shuttles to the cytoplasm when required to act on cytoplasmic targets, such as during inhibition of insulin signaling [7]. In contrast, *sirt2* is cytoplasmic. It deacetylates tubulin microtubules [8] and transcription factors those shuttle from the cytoplasm to the nucleus [9]. *Sirt3*, *sirt4* and *sirt5* are active in the mitochondria [10] by participating in the regulation of Adenosine Triphosphate (ATP) synthesis, metabolism, apoptosis and intracellular signaling [11]. Among them, *sirt3* may be moved between the nucleus and mitochondria under cellular stress [2]. *Sirt6* is a nuclear protein, although it is also present in the endoplasmic reticulum, where it deacetylates TNF-α [12]. *Sirt7* is a nuclear protein that is mostly expressed in the nucleolar regions [13].

**Table 1:** The location and enzymatic catalytic activity of sirtuins.

Sirtuin	Class	localization	Enzymatic activity
**Sirt1**	I	Nuclear/Cytosolic	Deacetylase,Deacylase
**Sirt2**	I	Nuclear/Cytosolic	Deacetylase, Deacylase
**Sirt3**	I	Mitochondrial/ Nuclear/Cytosolic	Deacetylase, Decrotonylase
**Sirt4**	II	Mitochondrial	Deacetylase, ADP-ribosyltransferase, Lipoamidase, Deacylase
**Sirt5**	III	Mitochondrial/ Nuclear/Cytosolic	Deacetylase, Desuccinylase, Demalonylase, Deglutarylase
**Sirt6**	IV	Nuclear/Cytosolic	Deacetylase,Demyristoylase, ADP-ribosyltransferase, Deacylase
**Sirt7**	IV	Nucleolar/nuclear	Deacetylase, Desuccinylase

## Overview of sirtuins functions

Sirtuins belong to the class III histone deacetylases (HDACs) [14].The sirtuin family shares a highly conserved catalytic domain, and exerts NAD^+^ -dependent protein deacylase and/or ADP ribosyltransferase activities [15, 16]. However, as shown in Table 1 & Table 2, the sirtuin family members differ from one another with respect to catalytic activities, subcellular localization, protein targets, and biological functions [17].

Studies indicate that, from yeast to humans, sirtuins contain a highly conserved catalytic core domain formed by 275 amino acids, and N,C-terminal extensions which is variable in length and sequence, and can affect the binding with interacting partners, mediate interactions with other sirtuin forms, and direct cellular localization. In addition to their well-studied roles as lysine deacetylases, certain sirtuins can also remove other acyl modifications from lysine residues, including propionyl, butyryl, malonyl, succinyl and the lengthy fatty-acid derived myristoyl and palmitoyl groups [18-22]. In addittion, sirtuins possess NAD^+^-dependent deacetylase, deacylase, desuccinylase, demalonylase, deglutarylase, ADP-ribosyltransferase activities, etc. and regulate many processes in vivo, including metabolism, DNA repair, metastasis, apoptosis, translation, promoting longevity and protecting against cancer via altering substrate activity, localization, stability and protein-protein interactions [23].

*Sirt1* is the closest to yeast *Sir2* in terms of sequence and enzymatic activity and is also the most extensively studied mammalian sirtuin at present. *Sirt1* deacetylates a diverse array of cellular proteins, including histones, transcription factors, DNA repair proteins, autophagy factors, and others, like FOXO3a, PPARα, PGAM-1, SREBP1, FXR, PGC-1α,NF-κB, etc [24] to modulate metabolism, stress responses, and other cellular processes [25]. *In vitro, sirt1* possesses deacylase activity, although the functional significance of this activity *in vivo* remains unclear [26].

*Sirt2* mainly functions in mitosis. *Sirt2* regulates mitotic progression by controlling the activity of the anaphase-promoting complex/cyclosome. When DNA damage emerges, *sirt2* may halt cell division, effectively guarding the cell against erroneous replication. *Sirt2* also plays an important role in controlling the cell cycle. In fact, an increase in *sirt2* activity significantly delays cell cycle progression [27]. In addition, the overall effect of *sirt2* upregulation on carbohydrate and lipid metabolism is similar to that of *sirt1*, promoting gluconeogenesis through deacetylation of phosphoenolpyruvate carboxykinase (PEPCK) [28] and inhibiting adipocyte differentiation through deacetylation of FoxO1 [9, 29]. Furthermore, *sirt2* also has anti-inflammatory effects [30].

*Sirt3* is a mitochondrial enzyme, and it deacetylates and activates mitochondrial enzymes to regulate diverse mitochondrial functions, such as ATP production, reactive oxygen species (ROS) management, β-oxidation, ketogenesis, and cell death [31]. The metabolic actions of *sirt3* on carbohydrate and lipid metabolism are similar to those of *sirt1* (e.g., stimulation of gluconeogenesis, inhibition of lipogenesis, activation of fatty acid oxidation, and some neuroprotective actions) [32]. Furthermore, *sirt3* has also been related to adaptive thermogenesis because of its regulation in both white and brown adipose tissue by caloric restriction (CR) and cold exposure [33].

Unlike the other sirtuins, *sirt4* is a mitochondrial sirtuin lacking *in vitro* deacetylase activity [34]. Biologically, *sirt4* functions in many important processes, particularly in glutamine and fatty acid metabolism [35]. *Sirt4* is also thought to regulate ATP homeostasis. *Sirt4* improves the efficacy of ATP synthesis by inhibiting the oxidative phosphorylation uncoupler ANT2 [36].

**Table 2:** The substrates/targets and functions of sirtuins.

	Substrates/targets			Function
	Modification	Activation	Inhiibition	
**SIRT1**	H3K9ac, H3K26ac, H3K16ac, H1K26, H1K9, H3K56, H3K14, H4K16, α-tubulin, p53	Suv39h1, N-Myc, ER, Sirt6, ADAM-10, LKB1, AMPK, NBS1, XPA, MnSOD, WRN, Ku70, FOXO1/3, PGC-1a, PPARα, FXR	NF-KB, p300, p66^shc^, mTOR, HIF-1a, TNF-1a, Histone acctylation, SREBP-1c	Glucose metabolism, fatty-acid and cholesterol metabolism, differentiation, insulin secretion, and neuroprotection, stress responses, DNA repair, vascular protection and other cellular processes
**SIRT2**	α-tubulin, H3K56ac, H4K16ac,	FOXO, c-Myc, G6PD, PEPCK	NF-KB, p53, FoxO1	Cell-cycle control, carbohydrate and lipid metabolism, tubulin and transcription factors deacetylation and anti-inflammatory
**SIRT3**	H3K9, H4K16, H3K56ac, H4K14ac,	Ku70, Mn-SOD, FOXO3a, DH2, FAO, GDH, complexI/III, IDH2	p53, HIF-1a, Ros, lipogcncasis	regulation of mitochondrial enzymes deacetylation, ATP production, reactive oxygen species (ROS) management, b-oxidation, ketogenesis, cell death, and carbohydrate and lipid metabolism
**SIRT4**			GDH, AMPK, ROS, PDH	Insulin secretion, glutamine and fatty acid metabolism and regulate the ATP homeostasis
**SIRT5**	CPS1	SOD1	GLS	Urea cycle, regulation of ATP synthesis, metabolism, apoptosis and intracellular signaling, regulation of ammonia detoxification, fatty acid oxidation
**SIRT6**	H2BK12, H3K9ac H3K56ac, WRN	FOXO, PARP1, CtIP, P53, DNA-PKcs, CCNDBP1	NF-KB, RELA, TNF-a, IGF-1, HIF-1a, Myc, c-Jun, PGC-1a, GCN5	Telomeres and telomeric functions, DNA repair, metabolic homeostasis, inflammation, stress responses, and genomic stability
**SIRT7**	H2A, H2B, H3 (H3K18)	FOXO	RNA-POLY-merase, HIF-1a/2a	regulates the transcription of rDNA and mediate histone desuccinylation

*Sirt5* is a mitochondrial enzyme [20]. It is now known that newly discovered PTMs removed by *sirt5* can regulate the activity of enzymes affecting the redox status of cells and energy utilization, but we have just started to learn about the exact influence of *sirt5* on these pathways. In addition to the deacylase activities of *sirt5*, recent studies have suggested that *sirt5* can mediate protein desuccinylation, demalonylation, and deglutarylation [21, 37, 38].

*Sirt6* deacetylates specific cellular targets: H3K9Ac and H3K56Ac, the DNA repair factor CtIP, and the acetyltransferase GCN5 [39]. Recent studies have shown that *sirt6* also has a deacylase activity [19]and interacts physically with some non-histone proteins - not only through deacylation, but also through direct physical interaction (PIA), inhibition of their binding at the target gene promoters (IATGP) and destabilization of their binding at the target gene promoters (DATGP)[40]. Through these functions, *sirt6* plays essential roles in metabolic homeostasis, inflammation, stress responses, and genomic stability [41].

*Sirt7* is the only sirtuin localized to the nucleolus and is a component of the RNA polymerase I (Pol I) transcriptional machinery. By interacting with RNA Pol I and histones, *sirt7* regulates the transcription of rDNA in mammalian cells [42]. In addition, *sirt7* can mediate histone desuccinylation [43].

## Sirtuins and aging

Ageing is a conserved phenomenon across all species and imposes an ever-increasing risk of dysfunction and death in older organisms. Growing evidences have shown that sirtuins are essential factors those delay cellular senescence and extends the organismal lifespan through the regulation of diverse cellular processes. Therefore, in the review, we summarize the evidences and controversies regarding the roles of different sirtuins on aging and lifespan extension, and systematically elucidate the functions and pathways of sirtuins on aging and lifespan extension.

The link between sirtuins and longevity was first established 20 years ago in yeast, in which the complex of *Sir2/3/4* extended the replicative lifespan of S. cerevisiae [44, 45].Research interests increased after a report showed that extra copies of *Sir2*, a member of sirtuins in budding yeast Saccharomyces cerevisiae, extended the lifespan by 30% by preventing the formation of extrachromosomal DNA circles [3]. Subsequently, more and more research shows that sirtuins can regulate longevity in numerous lower organisms, especially yeast *Sir2* and its homologues, which extend the lifespan of budding yeast *S. cereviseasae*, worms *C. elegans*, fruit flies D. melanogaster, and mice [46-48]. So far, The prolongevity effect of *sir2* has been confirmed in higher organisms, while the mechanisms of exerting prolongevity effects are different from that in yeast, including changes in mitochondrial function and biogenesis, suppression of inflammation, and regulation of genomic stability [49].

Though several reports have challenged this theory, sirtuins have long been recognized as regulators of ageing, and overexpression of some sirtuins has been shown to extend lifespan in several organisms. The suppression of cellular senescence by sirtuin is mainly mediated through delaying the age-related telomere attrition, sustaining genome integrity and promoting DNA damage repair [50]. According to reports, *sirt1* deacetylates histones H3, H4 and H1 and more than 50 non-histone proteins, including DNMT1, transcription factors and DNA repair proteins [2]. *Sirt1* and *sirt6* were shown to be recruited to the damaged sites and promote DNA repair through deacetylating the repair proteins such as poly (ADP-ribose) polymerase (PARP)-1, Ku70, NBS, and Werner (WRN) helicase [12, 51-53]. In mammals, sirtuin upregulation can work in a context-, tissue-, and particular sirtuin-dependent manner [54], and not all laboratories have managed to repeat the initial life span extending effect of sirtuins upregulation [55, 56].In addition, sirtuins are found to especially interact with all the major conserved longevity pathways, such as AMP-activated protein kinase (AMPK), insulin/IGF-1 signaling (IIS), target of rapamycin (TOR), and forkhead box O (FOXO). Of these, FOXO transcription factor is the most fascinating target of sirtuin. In *C. elegans*, the extension of lifespan by elevation of sir-2.1 was shown to be dependent on daf-16, the homologue of FOXO in worms [57-59]. Considering that FOXO is a major component in the IIS cascade to promote lifespan extension and stress resistance, several evidences have reported the association of the IIS pathway with the prolongevity effect of sirtuin. In *C. elegans*, the deletion of sir-2.1 had no effect on the lifespan of a long-living daf-2 mutants [60]. In mammals, the relationship of IIS and sirtuin has also been well investigated. *Sirt1* is reported to play a crucial role in metabolic homeostasis and IIS [61, 62]. AMPK signaling belongs to the protein kinase family and restores cellular energy levels. Increased AMPK activity is known to extend the lifespan of some model organisms. The mutation of AMPK (aak-2) in *C. elegans* abrogated the lifespan extension by sir-2.1 expression [63], indicating that AMPK also contributes to the sirtuin-induced lifespan extension. *Sirt1* activates AMPK through the direct deacetylation of LKB1, a regulator of AMPK [64]. In addition, AMPK contributes to the prolongevity effect of IIS, suggesting that these longevity pathways intricately cross-talk with each other. Apart from these, several other molecules are also reported to mediate lifespan extension by sirtuin overexpression, including 14-3-3, kat-1, hcf-1, and cts-1 in *C. elegans*. In addition, a study of mutant screening reported that loss-of-function mutations of ketoacyl thiolase (kat-1) resulted in premature aging and fully suppressed the lifespan extension exerted by overexpression of sir-2.1 [65]. Also, host cell factor-1 (hcf-1), a nuclear co-repressor of FOXO, was shown to act downstream of sir-2.1 to modulate the lifespan in C. elegant [66]. Furthermore, mitochondrial regulators such as cts-1 and fzo-1, and the mitochondrial unfolded protein response (UPRmt) gene hsp-6, were reported to be increased by sir-2.1 overexpression, and the knock-down of UPRmt regulator ubl-5 using RNAi almost completely suppressed the lifespan extension by sir-2.1 overexpression [67]. A more detailed description of the role about different sirtuins on aging and lifespan extension, and the signaling pathways are listed in Table 3.

**Table 3:** Functions and signaling pathway of sirtuins in aging.

	Sirtuin	Functions in aging	Regulatory factor in signal pathway	
			Positive	Negative
**Yeast**	*Sir2*	Replicative lifespan extension Cell cycle arrest	NAD+, mTOR, PKA, Sch9	
**C. elegans**	sir-2.1, sir-2.2, sir-2.3, sir-2.4	Lifespan extension, Stress resistance	mTOR, HLH-30, PHA-4, NHR-62, WWP-1, KLF-1, EGL-9, DAF-16, AAK-2, HSF-1, FOXA, DAF-2, IGF-1, SKN-1, SAMS-1, RAB-10, DRR-1, DRR-2	UNC-51, ATG1, ULK1, BEC-1, ATG6, Beclin1, VPS-34, ATG-18, Wipi, ATG-7, ATG-8, IGG-1, HIF-1, TOR, RHEB-1, INS-7
**Drosophila**	dSir2, Sirt4	Lifespan extension	FOXO,4E-BP	INR, p53, mTOR/S6K pathway
**Mammal**	sirt1	Lifespan extension, DNA repair, Cell cycle arrest, Cellular senescence	eNOS, Erβ, FOXO3	PAI-1, p53, p16^INK4a^, NF-KB, p66^5hc^, LKB1, Ciclin D1, mTOR/S6K pathway
	sirt 2	Cell cycle regulation	BubR1, PPP, NAD+, FOXOs, Mn-SOD	Wnt, AKT, NF-KB,
	sirt 3	Mitochondrial function, Oxidative stress, Centenarian-linked SNPs	FOXO3A, Mn-SOD	NF-KB, MAPK/ERK, P13K/AKt
	sirt 4	Fatty acid oxidation, Apoptosis		NF-KB, Bax, Caspase, GDH, AMPK
	sirt 5	Fatty acid oxidation, Oxidative stress		BCL, GDH, PDH
	sirt 6	Lifespan extension, DNA repair, Genome stability, Telomere maintenance	Nrf2, eNOS, IGF/AKt, p53	P21^Cip1/Waf1^, NF-KB, ICM-1, PAI-1,
	sirt 7	Epigenetic regulation, Stress resistance, Apoptosis	FOXO3	Myc, HIF-1a/2a, p53,

Among the mammalian sirtuins, *sirt1* has been the most extensively characterized for its role in aging. Although much of the attention has gone to *sirt1* and its protective effects against the onset of chronic diseases, its effect on longevity remains unconvincing. Sirtuins other than *sirt1* are also reported to exert a prolongevity effect. Recently, *sirt2* has been found to be a key modulator of ageing, and it extends lifespan in the BubR1 mice model[68]. Additionally, *sirt3* is the only sirtuin that has been shown to be associated with human aging; some (but not all) studies have linked polymorphisms in the *sirt3* genomic locus to survival in elderly individuals [55, 56, 69, 70]. However, no pan- or tissue-specific transgenic animal models overexpressing *sirt3* to determine whether *sirt3* overexpression confers lifespan extension or protects against age-associated pathologies have been described in the literature currently, and some newer studies failed to confirm these correlations in other populations [56, 70]. In contrast, recent work on *sirt6* suggests that this sirtuin might hold the most potential for actual life-span extension [47]. Loss of *sirt6* causes severe metabolic defects and rapid aging [71]. In addition, global *sirt7* depletion contributes to premature ageing, especially in the backbone, white adipose tissue and the heart [72, 73].

However, it is clear that we are just starting to appreciate the importance of identifying specific processes in aging regulated by the different members of the sirtuin family in a tissue-, cell type-, and gene specific context. Thus, identifying these processes might be necessary to gain a better understanding of their role and fill in the current knowledge gaps in the field.

## Sirtuins and ageing-related diseases

Although there has been emerging debate on the role of sirtuins in ageing and lifespan extension, mounting evidence suggests that sirtuins are indeed the critical modulators of ageing and aging-related diseases via different signalling pathways. The human sirtuin isoforms, *sirt1*-7, are considered attractive therapeutic targets for ageing-related diseases listed in Table 3, including diabetes, metabolic syndrome, cardiomyopathies, non-alcoholic hepatic steatosis, hyperinsulinism-induced dyslipidaemia, chronic inflammation, neurodegenerative diseases, and some types of cancer [74, 75]. Here, we summarize and discuss some of the recent discoveries in sirtuins biology and their functions in age-related diseases. Table 4 lists some age-related diseases in elderly population

## Sirtuins and neurodegenerative disease

Although the body’s organ systems experience a general decline in function with age, perhaps the most emotionally and physically devastating decline is associated with the CNS. Impaired CNS function, with its effects on cognition, memory, hearing, balance, and motor control, can lead to a rapid loss of quality of life. In recent years, the risk of neurodegenerative disease (e.g., Alzheimer’s disease (AD), Parkinson’s disease (PD), Huntington’s disease (HD), and others) has increased sharply with age [76]. Several studies have revealed the important roles of sirtuins in neuronal development and neurodegenerative disease [77, 78]. Though the ability of sirtuins to ameliorate CNS-specific disorders is still a very inchoate area of investigation, the initial findings offer significant promise.

Progress in understanding the neurobiological benefits of *sirt1* has been focused on animal models of different human CNS diseases. In mouse models of Alzheimer’s disease, brain-specific knockout of *sirt1* caused a significant elevation in β-amyloid plaques and reactive gliosis [79]. *Sirt1* also conferred neuroprotection in three different mouse models of Huntington’s disease [80, 81]. Brain-specific deletion of *sirt1* exacerbated the neurotoxicity associated with mutant huntingtin protein, whereas overexpression of *sirt1* attenuated the toxicity. In addition, in a mouse model of Parkinson’s disease, overexpression of *sirt1* reduced α-synuclein aggregates, reduced gliosis, and attenuated lethality [82]. In a mouse model of injury-induced axonal degeneration, NAD^+^ biosynthesis and its support of *sirt1* activity were shown to be essential in preventing axonal loss following axonal transection [83, 84].

Inhibition of *sirt2* by pharmacological and genetic means in invertebrate and cell culture models has suggested potential neuroprotective benefits [85]. Unfortunately, *sirt2* knockout mice lacked a remarkable phenotype related to neurodegeneration [86], calling into question the effect of *sirt2* on neuronal health in mammals.

Less data is available for other sirtuins. It has been found that short-term AD treatment with extracellular Aβ1-42 oligomers enhanced the expression of the *sirt4* gene, but prolonged treatment affected all three mitochondrial isoforms (*sirt3* to *sirt5*), suggesting that links between APP/Aβ and sirtuins might be more complex, and possibly reciprocal [87].

**Table 4:** Some age-related diseases in elderly population.

	Aging and age-related disease
**Neurodegenerative diseases**	Presbyophrenia, Huntington’s disease, Parkinson’s disease, Alzheimer’s disease, Amyotrophic lateral sclerosis
**Cardiovascular and cerebrovascular diseases**	Hypertension, Coronary disease, Cardiomyopathies, Elder valvular heart disease, Arrhythmia, Cardiac failure, Cerebral infraction, Atherosclerosis
**Metabolic related diseases**	Diabetes, Metabolic syndrome, Osteoporosis, Hyperinsulinism-induced dyslipidaemia, Uarthritis, Non-alcoholic hepatic steatosis,
**Others**	Scapulohumeral periarthritis, Chronic bronc, Chronic inflammation, Cancer

## Sirtuins and cardiovascular diseases

In addition, aging results in well-defined phenotypic changes which lead the cardiovascular system to develop diseases even in the absence of traditional risk factors [88]. Sirtuins have been associated with vascular diseases in humans, such as carotid plaques [89], carotid intima media thickness [90], arterial stiffness [91], and plaque area and morphology [92].

Whole body augmentation of *sirt1* activity confers resistance to many cardiovascular sequelae associated with metabolic syndrome. Study indicated that endothelial *sirt1* age-dependent depletion or its inactivation is a frequent companion of many cardiovascular diseases (CVDs) [93]. *Sirt1* is highly expressed in endothelial cells, and activation of *sirt1* in endothelial tissues may be beneficial in the protection of endothelial cell function with age [94]. Conversely, *sirt1* insufficiency results in greater foam cell formation and atherosclerotic lesioning [95]. Age-related loss of *sirt1* protein expression in human VSMC correlates with a loss of capacity for vascular repair, diminished stress response, and increased senescence [96, 97]. *Sirt1* appears to counteract atherosclerosis by inhibiting VSMC hypertrophy [98] and neointima formation and protecting against DNA damage, medial degeneration, and hypertension [99, 100]. In summary, *sirt1* acts as a cardioprotective molecule that protects from aging, induces resistance against hypertrophic and oxidative stresses, inhibits cardiomyocyte apoptosis, and regulates cardiac energy metabolism [101, 102]. However, the role of *sirt1* on longevity and human cardiovascular diseases is not fully convincing, so further studies are needed.

Hashimoto-Komatsu et al. showed that *sirt2* mediates microtubule reorganization induced by Ang II and cyclic stretch in endothelial cells, suggesting that *sirt2* is a key regulator of endothelial remodeling [103]. However, *sirt2* knockout mice have no cardiac abnormalities. Thus, further studies are warranted to precisely define the role of *sirt2* in cardiac contractile function under physiological and pathological conditions. *Sirt3* is necessary to prevent mitochondrial dysfunction and cardiac hypertrophy during ageing [104, 105]. Overexpression of *sirt3* blunts cardiac hypertrophy by decreasing oxidative stress via upregulation of endogenous antioxidants (like Mn-SOD2 and catalase). *Sirt4* is found to be specifically enriched in the heart, kidney, brain, and liver. Furthermore, *sirt4* can also negatively modulate insulin secretion, fatty acid oxidation, and mitochondrial gene expression in cardiomyocytes and the liver, although the mechanism remains elusive and in vivo data are actually absent [106]. Variants in *sirt5* gene have been found to be associated with the risk of carotid plaque development [96]. In addition, Liu et al. have demonstrated in vivo that *sirt5* plays a critical role in regulating cell viability in cardiomyocytes [107]. Future studies are required to investigate the correlations between *sirt5* level and on the effect of *sirt5* on various pathologic pathways of CVDs.

Based on the striking phenotype of *sirt6* knockout mice, which are predisposed to accelerated senescence, significant researches have shown in vivo that *sirt6* can also regulate cardiac hypertrophy and age-related cardiovascular alterations [108, 109]. Among nuclear sirtuins, *sirt1* and *sirt6* play an important role in prevention and delay of CVDs [110]. In fact, *sirt6* expression blocks the development of cardiac hypertrophy and heart failure [111, 112], whereas some data suggested that *sirt1* promoted cardiomyocyte hypertrophy [113]. To date, the molecular events through which *sirt6* exerts a protective role at cardiovascular level, regulating the endothelial cell and cardiomyocyte response to stress, reducing oxidative stress and hyperglycemia, are still unclear. Vakhrusheva et al. have demonstrated that *sirt7* plays an important role in preventing progressive functional deterioration of the heart [73]. *Sirt7* deletion leads to various pathological changes in the heart, which further aggravates with age, including heart hypertrophy, fibrosis, lipofuscin accumulation and inflammatory cardiomyopathy.

However, there are many unsolved issues regarding the function of sirtuins at cardiovascular level, and undoubtedly, more work is needed to understand the role of the different sirtuins in cardiac and vascular cell biology before they can be considered as a valuable therapeutic target against age-related cardiovascular diseases.

## Sirtuins and metabolic diseases

Furthermore, declines in basal metabolic rate and physical activity contribute to an elevated incidence of insulin resistance, obesity, and metabolic syndrome with age [114]. Sensing of the metabolic state and regulation of the sirtuin function and expression are critical components of the metabolic machinery. Thus, activation of pathways that restores insulin sensitivity and improves the utilization of glucose and fatty acids would be of benefit in stemming the pathologies associated with age-related metabolic dysfunction [115]. In the next review, we summarize an overview and update on the function of different sirtuin in metabolism, and further touch the correlation between each sirtuin and disorders of metabolism.

Many studies have indicated that *sirt1* is an important target for mitigating metabolic dysfunction. *Sirt1* is directly involved on metabolic pathways such as lipogenesis, stimulation of fatty acid β-oxidation, and gluconeogenesis. Its overexpression is thought to be beneficial and generates phenotypes in mice similar to calorie restriction conditions. All the major mitochondrial processes including the krebs cycle, the fatty acid metabolism, the antioxidant response, the amino acid catabolism, and so on, are regulated by the balance of N"-lysine acetylation/deacetylation. Several transgenic models have shown that heightened *sirt1* activity protects against the metabolic derangement associated with obesity [116]. *Sirt1* and *sirt1* activators can prevent and reverse insulin resistance and diabetic complications, and have been proven to be promising therapeutic targets for type 2 diabetes (T2D) [117-119]. In addition, the protective effects of *sirt1* may occur through attenuation of inflammatory responses, as *sirt1* overexpression mitigates HFD-induced hepatic steatosis and adipose tissue specific inflammation [120, 121].

Compared to *sirt1*, *sirt2* is abundant in adipocytes. Current evidence suggests a role for *sirt2* in regulating adipose tissue development and function. *Sirt2* activates the PEPCK via deacetylation and enhances gluconeogenesis during times of glucose deprivation [122]. Meanwhile, recent studies have proposed that, with regard to insulin sensitivity, *sirt2* may act in opposing roles in different tissues [123]. Thus, *sirt2* activation may prove to be protective against obesity, and its role in metabolic homeostasis deserves further exploration.

*Sirt3* may regulate cellular energy status both at transcriptional level in the nucleus and by posttranscriptional mechanisms in mitochondria, and its expression is higher in metabolically active tissues including brain, liver, heart, brown adipose tissue and skeletal muscle [124]. *Sirt3* functions by activating important enzymes during CR, such as 3-hydroxy-3-methylglutaryl- CoA synthase 2 for generation of ketones [125] and long chain acyl-CoA dehydrogenase for the oxidation of long-chain fatty acids [126]. *Sirt3* also activates glutamate dehydrogenase (GDH), facilitating gluconeogenesis from amino acids[127]. In addition, *sirt3* indirectly destabilizes the transcription factor HIF1α and subsequently inhibits glycolysis and glucose oxidation [128]. Intriguingly, recent studies have shown that *sirt3* levels in pancreatic islets are reduced in patients afflicted with type 2 diabetes [129], and *sirt3* overexpression in pancreatic β-cells promotes insulin secretion and abrogates endoplasmic reticulum (ER) stress that is connected to β-cell dysfunction and apoptosis [130].

In contrast to *sirt3*, hepatic *sirt4* expression declines slightly during caloric restriction (CR) and increases in genetic models of diabetes [34, 131, 132]. Little is known about the physiological relevance of *sirt4* and its role in metabolism. *Sirt4*, initially reported as a unique ADP ribosyltransferase, appears to blunt insulin secretion by reducing GDH activity [133]. In addition to GDH, a diverse range of *sirt4* targets are identified in the regulation of insulin secretion, including ADP/ATP carrier proteins, insulin-degrading enzymes, ANT2 and ANT3 [133]. Recently, Haigis and coworkers showed that *sirt4* promoted lipid synthesis and inhibition of fatty acid oxidation by deacetylation of malonyl CoA decarboxylase [134].

In contrast to other sirtuins, *sirt5* displays deacetylase and NAD^+^ dependent demalonylase and desuccinylase activities. *Sirt5* facilitates glycolysis by demalonylating the glycolytic enzyme glyceraldehyde phosphate dehydrogenase (GAPDH) [38]. A recent study proposed that *sirt5* might be positively correlated with insulin sensitivity, the biological significance of which still remains to be confirmed [135].

The indication of the connection between *sirt6* and metabolism was first provided by Mostoslavsky et al. who showed that *sirt6*-deficient mice had a loss of subcutaneous fat, lymphopenia and acute hypoglycemia [136]. Conversely, one recent study revealed that *sirt6* overexpression protects mice from diet-induced obesity, showing increased glucose tolerance and reduced fat accumulation [137]. In addition, *sirt6* may positively mediate glucose stimulated insulin secretion and overexpression of *sirt6* enhances insulin sensitivity in skeletal muscle and liver, which implicates that *sirt6* may act as an attractive therapeutic target for T2D [138].

In addition, *sirt7* knockout mice were resistant to glucose intolerance, and insulin sensitivity was improved in *sirt7* knockout mice receiving a high-fat diet [46], revealing a novel role for *sirt7* in glucose metabolism.

## Sirtuins and Cancer

Based upon statistics from the National Cancer Institute, 54% of all cancer cases occur in people over the age of 65. Cancer is recognized as an age-related disease and occurs in an exponentially increasing pattern in elderly individuals. In recent years, cancer has become a grim challenge for us. Cancer seems to be one of the biggest hurdles in our way to longer and healthier ageing lives.

Currently, accumulating evidence has shown that the aberrant epigenetic activation of sirtuin signaling pathways contributes to tumor carcinogenesis and may be potential therapeutic targets for future treatments and biomarkers in predicting prognosis in cancers [23, 139]. Interestingly, sirtuins seem to have a dual role in cancer [140]. On the one hand, some sirtuins help protect DNA from damage and oxidative stress, maintain genomic stability, limit replicative life-span, and protect organisms against cancer. On the other hand, some data suggest that the promotion of cell survival under stress conditions by sirtuins could be directly involved in tumorigenesis, as it would inhibit senescence and allow unchecked cell division [141].

The role of sirtuins, especially *sirt1* in carcinogenesis, appears to be opposing and complicated. Under normal conditions, in response to stress or to DNA damage, *sirt1* might promote cell survival via cell cycle arrest, DNA repair, or inhibition of apoptosis. If the stress signal becomes chronic or the levels of damage cross a certain threshold, *sirt1* could induce cell senescence and prevent carcinogenesis [116]. However, following chronic stress or DNA damage, the loss of a tumor suppressor or of any other checkpoint- related factor could cause an imbalance in these regulatory processes and induce *sirt1* overexpression beyond a critical limit. The aberrant overexpression of *sirt1* would in turn contribute to transformation and tumor formation by promoting cell growth and inhibiting apoptosis [142].

In fact, *sirt1* acts as a tumor keystone, and its level and action maintain a fine and delicate balance between suppression and promotion of oncogenesis. It is plausible that *sirt1* acts as a suppressor and then a promoter (or vice versa) depending on the stage and situation of tumourigenesis. The dual role of sirtuins in different cancers have has been summarized and shown in Table 5. Studies have shown that *sirt1* is upregulated in many human cancer cell lines, as well as in tissues collected from patients suffering from various types of cancer (e.g., lung cancer, prostatic cancer, colon cancer, breast cancer, ovarian cancer, leukaemia, neuroblastomas, osteo-sarcomas, etc.), suggesting that sirtuins might be cancer therapeutic targets and the *sirt1* inhibition in cancer cells could possibly inhibit cancer cell growth [143-145]. Other research, however, points to a tumor suppressive role for *sirt1*. Certain cancer types, such as oral squamous cell carcinoma (OSCC), restoration of *sirt1* levels in these cells results in inhibition of tumor growth [146]. In other cancer mouse models, *sirt1* can protect against the development of intestinal tumors in a β-catenin-driven colon cancer model [147], sarcomas, lymphomas, teratomas, and carcinomas arising from deletion of p53 [148], HFD-induced hepatocarcinomas [116], and age-associated spontaneous tumor development [116]. In addition, other studies reported only slightly elevated *sirt1* activity (in some thyroid cancers) or unchanged activity in some lung cancers, colon cancers, gastric cancers, urinary bladder cancers, and skin cancers [148].

**Table 5:** The dual role of sirtuins in different cancer.

	Oncogene/Tumor suppressor	Expression in tumor tissue	
		Up-regulated	Down- regulated
**SIRT1**	Both	lung cancer, prostatic cancer, colon cancer, breast cancer, ovarian cancer, leukemia, neuroblastomas, osteo-sarcomas, and non-melanoma skin carcinomas	early onset-mutant (BRCA1) breast cancer, beta-catenin-driven colon cancer model, sarcomas, lymphomas, teratomas, and carcinomas arising from deletion of p53, HFD-induced hepatocarcinomas, and age-associated spontaneous tumor development
**SIRT2**	Both	acute myeloid leukemia, pancreatic cancer, neuroblastoma, high-grade human HCC and prostate cancer	glioma, liver cancer, and esophageal and gastric adenocarcinomas
**SIRT3**	Both	oral cancer	40% of human breast and ovarian cancers
**SIRT4**	Tumor suppressor		small cell lung carcinoma, gastric cancer, breast cancer and leukemia
**SIRT5**	Both	non-small cell lung cancer, ovarian carcinoma	squamous cell carcinoma, endometrial carcinoma
**SIRT6**	Both	human skin squamous cell carcinoma and pancreatic, prostate and breast cancers	head and neck squamous cell carcinoma, colon, pancreatic, liver and non-small cell lung cancers
**SIRT7**	Oncogene	thyroid cancer, hepatocellular carcinoma, bladder cancer and colorectal cancer	

Similar to *sirt1*, *sirt2* is either a positive or a negative regulator of the tumorigenic process. The expression of *sirt2* has been found downregulated in several cancers, such as gliomas, breast cancer, head and neck squamous cell carcinoma, non-small cell lung cancer, and esophageal adenocarcinoma, and elevated in others, such as neuroblastoma, pancreatic cancer, and acute myeloid leukemia [149, 150].

The case of *sirt3* is more complex. Several studies indicate that *sirt3* is a tumor suppressor, mainly through mechanisms linked to oxidative response, energetic balance, and metabolic regulation [11, 151]. *Sirt3* expression is decreased in many human cancers, especially in 40% of human breast and ovarian cancers [108, 128]. However, in specific cancer types, *sirt3* turns out to be an oncogene and promote tumorigenensis [152, 153].

*Sirt4* mRNA levels were reduced in several human cancers, such as small cell lung carcinoma [154], gastric cancer [155], breast cancer and leukemia [156]. Lower *sirt4* expression is associated with shorter survival time in lung tumor patients [157]. A recent study has also shown that *sirt4* is a crucial regulator of stress resistance in cancer cells and *sirt4* loss sensitizes cells to DNA damage or ER stress [158].

Similar to other sirtuins, *sirt5* has been considered as a potential oncogene or tumor suppressor. *Sirt5* acts as a potential oncogene, and is overexpressed in non-small cell lung cancer [159] and ovarian carcinoma [160]. However, *sirt5* also emerged as a tumor suppressor in squamous cell carcinoma [161] and endometrial carcinoma [162].

*Sirt6* is thought to be a significant tumor suppressor protein (TSP), given its major role as a guardian of genome stability[163, 164]. *Sirt6* overexpression induces intense apoptosis in cancer cells but not in normal cells, which makes it an attractive ‘‘target’’ for future antineoplastic medications [165]. In addition, as with *sirt1*, *sirt6* plays both tumor suppressing and promoting roles. *sirt6* expression is downregulated in head and neck squamous cell carcinoma, and colon, pancreatic, liver and non-small cell lung cancers [161, 166, 167]. Conversely, increased *sirt6* expression has been reported in human skin squamous cell carcinoma and pancreatic, prostate and breast cancers, whose high expression suggests a poor prognosis and chemotherapy resistance[168-171].

Although *sirt7* has received comparatively less attention than other sirtuins, *sirt7* appears to have been regarded as a potential oncogene for its upregulation in all the cancer types studied so far, such as thyroid cancer, hepatocellular carcinoma, bladder cancer and colorectal cancer [172-174]. *Sirt7* can contribute to the maintenance of a transformed cell phenotype in tumor cells by suppressing the expression of some TSPs [15]. However, *sirt7* does not contribute to the initiation of carcinogenesis, which has been experimentally proven, as no correlation between *sirt7* upregulation in normal cells and their susceptibility to transformation has been found [6, 15].

## Activators and inhibitors of sirtuins

As the area of molecular science consolidates and advances, the sirtuin family members are gaining significance in human biology and disease. All of the above findings suggest that sirtuins show strong potential to become valuable predictive and prognostic markers for disease and therapeutic targets for the management of a variety of cancer types and other age-related diseases. Therefore, in the following review, we summarize the common activators and small molecule inhibitors of sirtuins in relieving aging symptoms and age-related diseases, which is shown in Table 6.

**Table 6:** Activators and inhibitors of sirtuins.

	Activators	inhibitors
**SIRT1**	Piceatannol, Resveratrol, SRT1720. SRT2104, 1,4-DHP derivative, SRT1460, SRT2183	Ex-527, ELT-11c
**SIRT2**	1,4-DHP derivative	AGK2, 3′-(3-fluoro-phenethyloxy)-2-anilino-benzamide, SirReal2, Compound 15e, UBCS0137, ELT-11c, Compound 28e, AEM2, TM, Chroman-4-one analogue, RK-9123016, NPD11033
**SIRT3**	Piceatannol, Resveratrol, 1,4-DHP derivative	Compound 8, ELT-11c, SDX-437
**SIRT4**		
**SIRT5**	Piceatannol, Resveratrol, UBCS039	
**SIRT6**	UBCS039	Compound 1
**SIRT7**		

## Small molecule activators of sirtuins

Dietary restriction (DR)/CR (the reduction of calorie intake without causing malnutrition) is the only known intervention able to increase the lifespan in many species, including yeast, fruit flies, nematodes, fish, rats, mice, hamsters and dogs [175, 176] and possibly even primates [177].

Since sirtuin is commonly believed to mediate the beneficial effects of CR, the activators of sirtuins are considered to mimic these beneficial effects and are hence attractive therapeutics for age-related diseases [178]. The search for molecules those activates sirtuins began more than a decade ago. These sirtuin-activating compounds (STACs) are mainly divided into two categories. One is the use of exogenous activators, the other is replenishment of the cellular NAD^+^ [75, 179].The first STACs were discovered for *sirt1* in 2003, and the most potent of which was resveratrol. This initial discovery was important because it proved that allosteric activation of sirtuins was possible [179]. Treatment with resveratrol and its derivatives brought some beneficial effects of *sirt1* induction without applying CR [180, 181]. Moreover, studies have shown that the sirtuin activator resveratrol has chemo preventive activity against various cancers, including leukemia, DMBA-induced mammary tumors in rats, skin cancer, and prostate cancer [182-184]. Following the discovery of resveratrol, a few researchers tried to find other selective activators [181]. High-throughput screening and medicinal chemical efforts have since identified more than 14,000 STACs from a dozen chemical classes, including several classes of plant derived metabolites such as flavones, stilbenes, chalcones, and anthocyanidins, which directly activate *sirt1 in vitro* [179]. Synthetic STACs include a number of agents such as imidazothiazoles (e.g., SRT1720) [185], thiazolopyridines (e.g., STAC-2), benzimidazoles (e.g., STAC-5), bridged ureas (e.g., STAC-9 ), cilostazol [186], paeonol [187], statins [188], hydrogen sulfide [189, 190] and persimmon [191]. All of these chemical classes activate *sirt1* by lowering the Km value of the substrate through a K-type allosteric activation mechanism[192, 193]. In addition, there are some natural anti-ageing compounds, such as quercetin, butein, fisetin, kaempferol, catechins and proanthocyanidins [194].

An alternative approach to activating sirtuins is to regulate NAD^+^ level by activating enzymes involved in biosynthesis of NAD or by inhibiting the CD38 NAD hydrolase [195-197].It has been known since 2003 that upregulation of the NAD salvage pathway, which recycles NAD^+^ from NAM, can extend lifespan and mimic calorie restriction in yeast [198, 199]. NAD-boosting molecules constitute a newer class of STACs those are gaining attention as a way to restore NAD^+^ levels in elderly individuals and potentially activate all seven sirtuins with a single compound. Examples of NAD-boosting molecules include NMN, nicotinamide riboside67 [200-203], and inhibitors of CD38 such as apigenin [196], quercetin [196] and GSK 897-78c [204]. In addition, targeting the enzymes that regulate NAD^+^ levels, such as CD38, CD157 and NAMPT, may also be worth exploring for their therapeutic potential. Moreover, malate dehydrogenase, MDH1, which is involved in energy metabolism and reduces NAD+ to NADH during its catalytic reaction, also plays a critical role in cellular senescence.

## Small molecule inhibitors of sirtuins

The inhibition of sirtuins has attracted more interest as a potential therapeutic anticancer strategy. Here, the fundamental principle of developing inhibitors is that they should have two characteristics: they should be potent and selective. The mechanism of a potent inhibitor is as follows: combining of the inhibitor and the sirtuin may be easier than that of Nε-acyl-lysine and the sirtuin, and the inhibitors may be processed by the sirtuin into a non-catalytic intermediate which can bind tightly to the active site of the sirtuin to prevent the normal binding and processing of the substrate [205]. A selective inhibitor should perfectly inhibit one certain sirtuin, for example *sirt2,* while not reacting with other sirtuins. Based on these 2 principles, there are some inhibitors already designed, such as NAM and thioacyllysine-containing compounds. However, there are still limits for such inhibitors.

Except for the mechanism-based inhibitor discussed above, there is another common way to develop an inhibitor for sirtuin, which is by chemical library screening. In this way, thousands of molecules can be screened. Of these screened molecules, there are 5 compounds potent and selective enough, including sirtinol and its analogues, splitomicin and its derivatives, indole derivatives, and tenovin and its analogues, to presumably work by noncovalently binding to the sirtuin active site and blocking substrate binding.

Compound 6 was found to be a highly potent inhibitor of the deacetylation reaction catalysed by *sirt1*. Compound 6 may have much less inhibitory effect on reactions catalysed by *sirt2* and *sirt3* compared with *sirt1*. SirReal 2 and Compound 7 were screened from an internal compound library, and were demonstrated to be potent *sirt2* inhibitors [206, 207]. Compound 8 is another potent and selective *sirt2* inhibitor. X-ray analysis showed that when this compound binds to the active domain of *sirt2*, it may form a rigid cyclic structure by intramolecular hydrogen bonding. Therefore, it may tightly bind with *sirt2*, highly potently and selectively inhibiting the activity of *sirt2* [205]. In addition, AK-7 is a brain-permeable selective *sirt2* inhibitor. It is neuroprotective *in vitro*, by reducing polyglutamine inclusions and cholesterol levels in neurons [208]. Tenovin-6, salermide and benzodeazaoxaflavin are small-molecule inhibitors of *sirt1* and *sirt2* [209, 210]. Suramin inhibits *sirt5* by binding into the B- and C-pockets of the NAD^+^ binding site as well as to the substrate-binding site [211]. In total, segmented inhibition or activation of sirtuin activity might confer therapeutic potentials in the future, mainly because of its double-edged sword effects in cancer cells.

In addition, Natural compounds present in the diet, classed as functional food/nutrients, are a great promise for health and longevity promotion and prevention of age-related chronic diseases [212] . Such compounds are nontoxic, easy to use and commonly available and could be included into a normal diet for long lasting supplementation. Several reports emphasized that dietary supplementation of polyphenols may protect against neurodegenerative, cardiovascular, inflammatory, metabolic diseases and cancer by enhancing *sirt1* deacetylase activity. However, in humans, the therapeutic and pharmacological potential of these natural compounds remains to be validated in clinical conditions.

## Implications and future perspectives

Since the discovery of the yeast *Sir2*, sirtuins have been focused on their functions in ageing and age-related disease. Accumulating studies have revealed that sirtuins exert profound protective functions against ageing-related pathologies and the degeneration of tissues and organs in elderlyindividuals. Their multivalent roles in blocking the development of ageing and ageing-related diseases further mark them as promising targets for developing interventions for several age-associated pathologies.

The intense efforts in the past ~15 years to develop isoform specific small molecule modulators of the sirtuins have yielded key insights into the pathophysiological roles of sirtuins and have seen great strides in understanding the enzymatic reaction mechanisms. Initial drug development efforts focused on *sirt1* and *sirt2* have yielded a number of potent *sirt2* inhibitors and *sirt1* activators, which have now been passed the first clinical trials, with evidence of safety and efficacy.

However, there is little standardization of the disease models currently used to study sirtuin therapeutic biology, leading to contradictory reports with limited cross-validation of the findings. There is still a long way to go for a molecule to be applied in treatment, with hurdles of regarding side effects, stability, and selectivity. The field of sirtuin modulators has clearly matured into an exciting path of drug development that holds the promise for treating common and rare diseases, with considerable unmet demand.
